# “Second victim” as a psychiatrist: a cross-sectional survey about consequences of patient suicide on European early careers psychiatrists and psychiatric trainees

**DOI:** 10.1192/j.eurpsy.2025.2360

**Published:** 2025-08-26

**Authors:** G. Longo, F. Santos Martins, I. P. Peyneshki, E. K. Duranté, M. Gostiljac, D. Cavaleri

**Affiliations:** 1 Department of Neurosciences/Department of Experimental and Clinical Neurosciences (DIMSC), Polytechnic University of Marche, Unit of Clinical Psychiatry, Ancona, Italy; 2 European Federation of Psychiatric Trainees (Research Working Group), Brussels, Belgium; 3 Centro Hospitalar Universitário S. João (CHUSJ), Porto, Portugal; 4 University Psychiatric Services, Old Age Psychiatry, Bern, Switzerland; 5 Center for Research in Epidemiology and StatisticS (CRESS), INSERM UMR 1153, Université Paris Cité, Paris, France; 6 University Clinical Center of Serbia, Clinic for Psychiatry, Belgrade, Serbia; 7 University of Milano-Bicocca , School of Medicine and Surgery, Monza, Italy

## Abstract

**Introduction:**

Suicide involves not only patients but also families and communities, causing long-lasting effects on those who “survive”. The term “second victim” is used to define people who experience significant distress after a patient suicide (PS). For mental health professionals, PS could be considered an “occupational hazard”.

**Objectives:**

To assess the impact of patient death on psychiatric trainees and early career psychiatrists (ECPs), comparing PS to other causes of death.

**Methods:**

Participants completed a socio-demographic section and a section about the experience of PS. Impact of event scale–revised version (IES-R) based on the last 7 days and the 7 days after the most recent patient death, Suicide Knowledge and Skills Questionnaire (SKSQ), the Impact of a Patient’s Suicide on Professional and Personal Lives Scale and the Maslach Burnout Inventory (MBI) were administered.

**Results:**

110 subjects were recruited from 23 European and 1 Asian countries. The mean age was 31.9 (SD=4.7). Most were trainees (66.4%, n=73), worked in a psychiatric ward (61.8%, n=68), and in general adult psychiatry (83.6%, n=92). Patient death was experienced by 51.8 % (n=57) of the participants. 17.3% (n=19) experienced a PS, 12.7% (n=14) experienced multiple PS, 13.6% (n=15) had patients who died both by suicide and other medical conditions, and 8.2% (n=9) had patients who died from other medical conditions. The most reported feelings were sadness, regret, guilt, helplessness and frustration. Among participants who experienced at least one PS, 89.7% (n=35) developed symptoms. The most common were increased awareness of risk (40.4%; n=19), low mood (34.0%; n=16), anxiety (32.6%; n=15) and lack of concentration (26.1%; n=12). 6.5% (n=3) experienced suicidal thoughts/passive death wishes, and 6.5% (n=3) received individual psychotherapy treatment for their symptomatology. Having experienced a patient loss influenced clinical practice in 33.3% (n=19) of the sample, with 10.5% (n=6) reporting the affliction of the ability to carry out clinical duties. 12.7% (n=14) considered changing careers, 10.5% (n=6) took sick leave, 57.8% (n=33) received helpful support from colleagues. However, 52.3% (n=30) felt they needed additional support. According to the total score of IES-R scored on the 7 days after the most recent patient death, 22.9% (n=11) of the sample who experienced at least one PS had a score indicating a risk of PTSD, compared to 22.2% (n=2) of participants who experienced other type of patient death. No difference in all scales was observed in those experienced PS rather than any other kind of patient death (p>0.05).

**Conclusions:**

Our results confirm that PS affects the mental health of ECPs and psychiatric trainees, and impacts their daily lives. A larger sample should be collected to strengthen our results and better characterize the impact of these events.

**Disclosure of Interest:**

None Declared

